# Systematic review and meta-analysis of *Coptis chinensis* Franch.-containing traditional Chinese medicine as an adjunct therapy to metformin in the treatment of type 2 diabetes mellitus

**DOI:** 10.3389/fphar.2022.956313

**Published:** 2022-09-08

**Authors:** Linlin Pan, Xin Zhai, Zhanhui Duan, Kun Xu, Guirong Liu

**Affiliations:** ^1^ Department of Traditional Chinese Medicine, Shandong University of Traditional Chinese Medicine, Jinan, China; ^2^ Department of Chinese Medicine Literature and Culture, Shandong University of Traditional Chinese Medicine, Jinan, China

**Keywords:** coptis chinensis franch, type 2 diabetes mellitus, systematic review, meta-analysis, curative effect

## Abstract

**Background:** In China, *Coptis chinensis* Franch. (Chinese name: Huanglian) prescriptions (HLPs) are prominent hypoglycemic agents used in glycemic control. However, the curative effect of HLPs as adjunctive therapies for type 2 diabetes mellitus (T2DM) has not been evaluated. Based on a systematic review and a meta-analysis, this study was conducted to assess the effects of HLPs combined with metformin as a reinforcing agent for T2DM.

**Materials and methods:** A total of 33 randomized controlled trials (RCTs) reporting on 2,846 cases concerning the use of HLPs in the treatment of T2DM were identified from the China National Knowledge Infrastructure (CNKI), Weipu (VIP), Wanfang, PubMed, Cochrane Library, and EMBASE databases. Primary outcomes included fasting blood glucose (FBG), 2-h postprandial blood glucose (2hPG), glycosylated hemoglobin, type A1c (HbA1c), fasting serum insulin (FINS), and homeostasis model assessment of insulin resistance (HOMA-IR). Secondary outcomes included total cholesterol (TC), triglyceride (TG), low-density lipoprotein cholesterol (LDL-c), high-density lipoprotein cholesterol (HDL-c), and gastrointestinal dysfunction (GD). Continuous data were expressed as mean differences (MDs) with 95% confidence intervals (CIs). The methodological quality of the included RCTs was assessed by Cochrane evidence-based medicine systematic evaluation. Statistical analysis was performed using the Review Manager and Stata software. The required information size and treatment benefits were evaluated by trial sequential analysis (TSA). The quality of evidence was rated using the Grades of Recommendation Assessment, Development, and Evaluation (GRADE) approach.

**Results:** The results revealed that HLPs are beneficial to improve the following: FBG (MD = −1.16%, 95% CI: −1.24 to −1.07), 2hPG (MD = −1.64%, 95% CI: −1.84 to −1.43), HbA1c (MD = −0.78%, 95% CI:−0.96 to −0.60), FINS (MD = −1.94%, 95% CI: −2.68 to −1.20), HOMA-IR (MD = −0.77%, 95% CI: −1.28 to −0.27), TC (MD = −0.70%, 95% CI: −1.00 to −0.39), TG (MD = −0.57%, 95% CI: −0.74 to −0.40), LDL-c (MD = −0.70%, 95% CI: −0.97 to −0.43), and HDL-c (MD = −0.21%, 95% CI: −0.32 to −0.10) for patients with T2DM. The funnel plot, Egger’s test, and trim-and-fill method indicated a moderate publication bias in the results. The TSA showed that the required sample size of HLPs in improving FBG, 2hPG, HbA1c, FINS, HOMA-IR, TC, TG, LDL-c, and HDL-c could sufficiently draw reliable conclusions. GRADE assessment revealed that the quality of the evidence for the effectiveness of HLPs in improving FBG was moderate, but the quality of evidence for 2hPG, HbA1c, FINS, HOMA-IR, TC, TG, LDL-c, and HDL-c was low, and for GD was very low.

**Conclusion:** The systematic review and meta-analysis suggested that HLPs were beneficial for achieving glycemic control. However, HLPs recommended for T2DM patients have yet to be confirmed because of the poor methodological quality of some trials. Therefore, more RCTs with multicenter and double-blind designs are needed to assess the efficacy of HLPs for patients with T2DM.

## 1 Introduction

Diabetes mellitus, which seriously endangers human health, is mainly caused by defects in insulin secretion and insulin action and is characterized by disorders of glucose metabolism. (Lin and Sun, 2010). An International Diabetes Federation survey predicted that patients with diabetes mellitus will exceed 645 million by 2045 (Carracher et al., 2018). Generally, more than 90% of diabetes mellitus patients have type 2 diabetes mellitus (T2DM). In addition to following diet and lifestyle guidelines, due to the significant hypoglycemic effect of metformin, it is often recommended to intervene with metformin in patients with T2DM (Sharma et al., 2015; Sanchez-Rangel and Inzucchi, 2017). However, due to the certain limitations of metformin in long term use, options from natural products are being searched to meet the need (Sharma and Prajapati, 2017). In recent decades, traditional Chinese medicine (TCM) and its active ingredients have become increasingly popular in Asian countries, and combined with metformin, is widely used as a reinforcing agent in glycemic control (Pang et al., 2018; Tian et al., 2019; Wu et al., 2019).

Ancient TCM theories effectively study a disease as a whole and propose that the pathogenesis of diabetes mellitus lies in damp-heat accumulation in the spleen and stomach (Tong et al., 2009). In classic TCM books, *Explanation of Materia Medica* (Chinese name: *Bencaojing Jizhu*) and *Tang Materia Medica* (Chinese name: *Tang Bencao*) clarified that the prescriptions containing *Coptis chinensis* Franch. (Chinese name: Huanglian) can effectively alleviate the symptoms of polydipsia, polyphagia, and polyuria (Tong, 2013). *Coptis chinensis* Franch. As a treatment for diabetes mellitus and related complications, also has a long history in Japan, Korea, Malaysia, Singapore, and India (Li et al., 2013; Sharma et al., 2021). Modern pharmacological investigations have indicated that some ingredients in *Coptis chinensis* Franch. such as berberine, jatrorrhizine, coptisine, palmatine, epiberbeine, and polysaccharides, exert significant therapeutic effects on multiple targets to improve islet function and regulate glucose metabolism (Fu et al., 2005; Chen et al., 2012; Wang et al., 2019). For example, alkaloids can help alleviate hyperglycemia by promoting glucose uptake (Yang et al., 2014), polysaccharides can produce antidiabetic activity via its antioxidative effect (Jiang et al., 2015), and berberine can improve insulin resistance by inhibiting the expression of tumor necrosis factor-α and free fatty acids (Huang et al., 2018).

Recent studies have indicated that Huanglian prescriptions (HLPs) contribute to enhancing insulin sensitivity, stimulating insulin secretion, protecting β-cells, and regulating glycometabolism disorders (Liu et al., 2010; Yu et al., 2012; Li et al., 2019). Therefore, either as monotherapy or adjunct therapy, HLPs are recognized as the most effective TCM antidiabetic prescriptions for T2DM in China. HLPs, such as Dahuang huanglian xiexin (DHHL) decoction, Gegen qinlian (GGQL) decoction, Huanglian ejiao (HLEJ) decoction, Huanglian jiedu (HLJD) decoction, and Huanglian wendan (HLWD) decoction, have been widely used as adjuvant therapies to metformin for glycemic control (Fan et al., 2017; Li et al., 2017; Song et al., 2022; Wang 2020; Zhou et al., 2022). However, to date, there is no large scale clinical evidence on the inhibitory effects of HLPs on T2DM. Also, no published reports can comprehensively evaluate the intervention and side effects of HLPs on glycolipids. Therefore, we included clinical randomized controlled trials (RCTs) for systematic review and meta-analysis to evaluate the effectiveness of HLPs as adjuvant therapies to metformin for patients with T2DM.

## 2 Materials and methods

This study followed the Preferred Reporting Items for Systematic Reviews and Meta-Analyses (PRISMA) guidelines, obtaining data from published trials.

### 2.1 Search strategies

All articles were searched using medical subject headings terms and free words in the China National Knowledge Infrastructure (CNKI), Wanfang, Weipu (VIP), PubMed, Cochrane Library, and EMBASE databases. The search period for the encompassed articles from the established time to 30 July 2022. Two authors (Xin Zhai and Linlin Pan) independently searched the related articles regardless of type and language. The following terms were used in English databases: [“Type 2 diabetes” or “Type 2 diabetes mellitus” or “T2DM” or “Non insulin dependent diabetes mellitus” or “Impaired fasting glucose” or “Impaired glucose tolerance” or “Xiaoke”] and [“Random allocation” or “Randomized controlled trial” or “Random” or “Randomized” or “Placebo” or “RCT”] and [“Huanglian”or “*Coptis chinensis* Franch.” or “Coptidis Rhizoma” or “Coptis chinensis” or “Rhizoma coptidis”]. The following terms were used in Chinese databases: [“Erxing Tangniaobing” or “Xiaoke” (T2DM) ] and [“Suiji duizhao shiyan” or “Mangfa” or “Anweiji” (RCT) ] and [“Huanglian”]. The search strategies are presented in detail in Supplementary Table S1.

### 2.2 Inclusion and exclusion criteria

The inclusion criteria were as follows: 1) *Participants*. Diagnosed with T2DM; 2) *Interventions*. Control group treated with metformin and experimental group treated using metformin incorporated with HLPs; 3) *Type of trials*. RCT; 4) *Outcomes*. Fasting blood glucose (FBG), 2-h postprandial blood glucose (2hPG), glycosylated hemoglobin, type A1c (HbA1c), fasting serum insulin (FINS), homeostasis model assessment of insulin resistance (HOMA-IR), total cholesterol (TC), triglyceride (TG), low-density lipoprotein cholesterol (LDL-c), high-density lipoprotein cholesterol (HDL-c), and gastrointestinal dysfunction (GD). The exclusion criteria were as follows: 1) Non-clinical intervention trials (animal research, cell research, review, protocol); 2) Patients diagnosed with other diseases; 3) Patients with other TCM medications, acupuncture, massage or moxibustion.

### 2.3 Literature selection and data extraction

Two authors (Linlin Pan and Xin Zhai) independently evaluated the title, abstract, and full texts of the articles. The articles that met the inclusion criteria were then selected. Inconsistencies were settled by discussion. Finally, important information from the included articles was extracted for analysis, including the name of the first author, year of publication, trial types, sample size, sex, age, course of the disease, interventions, and course of treatment.

### 2.4 Risk of bias

Linlin Pan and Xin Zhai independently evaluated the methodological quality of each trial by using the Cochrane risk-of-bias tool (Higgins et al., 2011). Disagreements were discussed and resolved by Guirong Liu. The criteria assessed were as follows: random sequence generation, allocation concealment, blinding of participants and personnel, blinding of outcome assessment, incomplete outcome data, selective reporting, and other biases. The risk of bias was rated as high, unclear, or low.

### 2.5 Data synthesis and analysis

RevMan (version 5.3) was used to perform statistical analysis. Continuous data were expressed as the mean difference (MD) with a 95% confidence interval (CI), and *p* < 0.05 was considered statistically significant. Heterogeneity was evaluated using the chi^2^ and I^2^ tests, and *p* < 0.10% or I^2^ > 50% was considered to have marked heterogeneity. The low-heterogeneity data (*p* > 0.10% or I^2^ < 50%) used the fixed-effect model, and the high-heterogeneity data (*p* < 0.10% or I^2^ > 50%) used the random-effects model. Sensitivity analysis was evaluated using various statistical methods. Publication bias was assessed by visual observation of the symmetry of funnel plots, Egger’s test (*p* < 0.05 indicates publication bias), and the trim-and-fill method.

Trial sequential analysis (TSA) was conducted to calculate the required information size (RIS) for meta-analysis and evaluate the intervention benefits on the basis of the accrued information size (AIS). The risk of a type I error was set at 5% with a power of 80%. The variance was calculated based on the data included in the trials, and the relative risk reduction was set at 20% (Wetterslev et al., 2017). The evidence for the intervention was considered reliable when cumulative Z-curves crossed sequential monitoring boundaries. The Grades of Recommendation Assessment, Development, and Evaluation (GRADE) approach was used to rate the quality of the evidence as high, moderate, low, or very low (Guyatt et al., 2008).

## 3 Results

### 3.1 Search results

**FIGURE 1:**
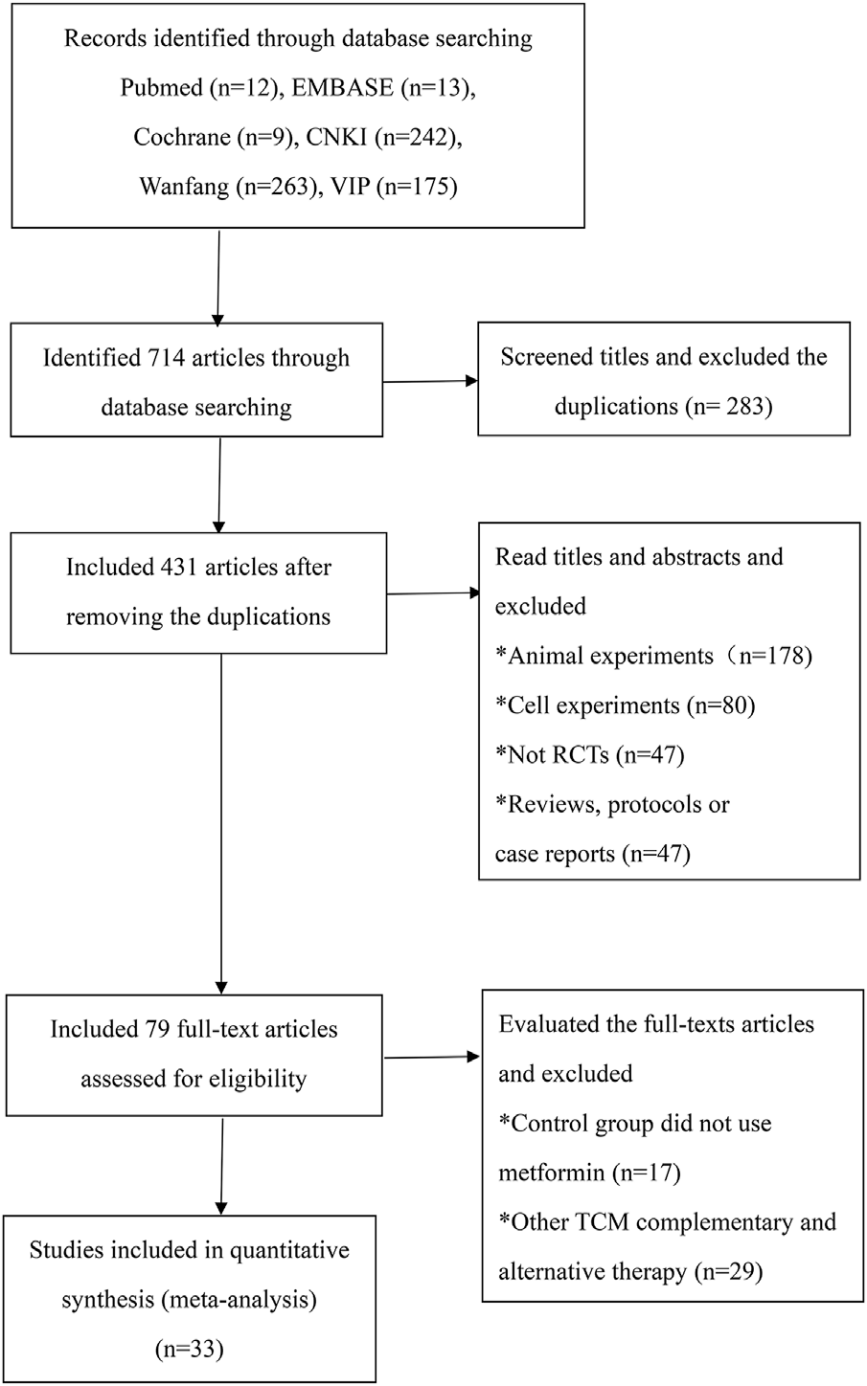
Flow diagram of the literature search process.

A total of 714 articles were identified in the initial database search (Figure 1). First, we used Endnote to exclude 283 duplicates, and the articles were decreased to 431. Second, we read the titles and abstracts and excluded animal experiment articles (*n* = 178), cell experiment articles (*n* = 80), reviews (*n* = 24), protocols (*n* = 8), case reports (*n* = 15), and non-RCT experimental trials (*n* = 47). Third, the trials using other TCM therapies (*n* = 29) and without metformin in the control group (*n* = 17) were excluded after reading the full text. Ultimately, 33 RCTs satisfying the inclusion criteria were identified (Yang and Wang, 2013; Zhang, 2014; Zhang et al., 2013; Zhou et al., 2022; Zou and Lao, 2016; Dong, 2017; Fan et al., 2017; Fu, 2017; Ji, 2017; Li et al., 2017; Liu, 2017; Liu, 2017; Xing et al., 2017; Chen et al., 2018; Ding, 2018; Pang et al., 2018; Song et al., 2022; Zhang et al., 2018; Feng, 2019; Jin et al., 2019; Wu et al., 2019; Xiong, 2019; Zhang, 2019; Zhang et al., 2019; Gao, 2020; Wang, 2020; Chen and Wang, 2021; Fu, 2021; Liu et al., 2021; Pan et al., 2021; Wang et al., 2021; Wang Y 2022; Wei et al., 2021).

### 3.2 Study characteristics

A total of 33 RCTs published from 2006 to 2022 were included in this study. The RCTs consisted of 2,846 patients with T2DM between 18 and 86 years of age (Table 1). All trials were single-center trials, and the detection time ranged from 2 to 24 weeks. A total of 1,437 patients in the experimental group underwent treatment using HLPs plus metformin, and 1,409 patients in the control group underwent metformin treatment. Among the 33 trials, three trials with 268 patients used DHHL decoction, seven trials with 551 patients used GGQL decoction, five trials with 438 patients used HLEJ decoction, eight trials with 885 patients used HLJD decoction, and ten trials with 704 patients used HLWD decoction (Table 2).

**TABLE 1:** Characteristics of the included studies.

References	Trial types	Sample size (E/C)	Sex (M/F)	Age (years) (E/C)	Course of disease (year) (E/C)	Interventions		Course of treatment
			EC			E	C	
Li et al. (2017)	RCT	76 (43/33)	Unknown	39-67 (Mean 53.2 ± 7.7)	Mean 5.3 ± 3.4	DHHL	M (50 mg ET, Tid)	12 weeks
Wu et al. (2019)	RCT*	86 (43/43)	(28/15)/(29/14)	Mean 52.6 ± 10.2/52.3 ± 9.7	Mean 2.6 ± 1.7/2.6 ± 1.6	DHHL	M (0.5 g ET, Tid)	Unknown
Zou et al. (2016)	RCT*#	106 (53/53)	(29/24)/(27/26)	Mean 53.69 ± 10.14/52.38 ± 10.03	Median 5-10	DHHL	M (50 mg ET, Tid)	24 weeks
Fan et al. (2017)	RCT	70 (35/35)	(21/14)/(19/16)	18-60 (Mean 36.4 ± 7.1/38.0 ± 6.5)	Mean 3.1 ± 1.7/3.4 ± 1.5	GGQL	M (0.85 g ET, Bid)	8 weeks
Fu (2017)	RCT*	66 (33/33)	(17/13)/(16/14)	18-60 (Mean 56.07 ± 8.25/57.50 ± 8.19	Mean 5.20 ± 2.09/5.60 ± 2.01	GGQL	M (0.5 g ET,Tid)	12 weeks
Jin et al. (2019)	RCT*	60 (30/30)	(16/14)/(18/12)	25-83(Mean 58.06 ± 3.14)/22-85 (Mean 57.98 ± 3.72)	Mean 3.52 ± 0.86/3.47 ± 0.91	GGQL	M (0.5 g ET, Qd)	8 weeks
Pang et al. (2018)	RCT#	90 (45/45)	(23/22)/(24/21)	41-72(Mean53.5 ± 8.2)/42-71(Mean54.1 ± 8.3)	Mean 5.5 ± 1.3/5.4 ± 1.1	GGQL	M (0.25 g ET, Tid)	8 weeks
Xiong (2019)	RCT*	100 (50/50)	(29/21)/(30/20)	40-70(Mean53.7 ± 7.7)/40-70(Mean53.5 ± 7.8)	Mean 4.85 ± 1.05/4.75 ± 1.10	GGQL	M (0.25 g ET, Tid)	8 weeks
Zhang (2019)	RCT	70 (35/35)	Unknown	35-70/36-71	Unknown	GGQL	M (0.25 g ET, Bid)	8 weeks
Zhang et al. (2018)	RCT*	95 (48/47)	(26/22)/(25/22)	Mean 51.3 ± 6.8/51.2 ± 7.3	Mean 5.4 ± 2.3/5.6 ± 2.1	GGQL	M (0.5 g ET, Tid)	8 weeks
Liu (2006)	RCT	76 (47/29)	(29/18)/(17/12)	45-63/41-65	1.5-16/1-16	HLEJ	M (0.5 g ET, Tid)	4 weeks
Liu et al. (2017)	RCT*	86 (43/43)	(30/13)/(28/15)	45-76 (Mean 65.4 ± 4.7)/45-75 (Mean65.1 ± 4.8)	Mean 6.8 ± 1.7/6.4 ± 1.5		M	
Gao (2020)	Unknown	66 (33/33)	(18/15)/(19/14)	58.62 ± 6.13/58.54 ± 5.49	Mean 6.02 ± 1.68/6.08 ± 1.70	HLEJ	M (0.5 g ET, Bid)	4 weeks
Wang (2020)	RCT*	90 (45/45)	(27/18)/(23/22)	Mean 51.4 ± 3.4/52.3 ± 5.1	Unknown	HLEJ	M (0.5 g ET, Tid)	15 days
Zhou et al. (2022)	RCT	120 (60/60)	(29/31)/(33/27)	22-75 (Mean 53.25 ± 11.47)/24-75(Mean 54.25 ± 10.85)	Mean6.45 ± 2.51/6.51 ± 2.44	HLEJ	M (0.5 g ET, Tid)	8 weeks
Ding (2018)	RCT	104 (52/52)	(22/30)/(23/29)	57-86 (Mean 69.0 ± 4.6)/57-85(Mean 69.2 ± 4.7)	Mean 4.3 ± 2.4/4.1 ± 2.5	HLJD	M (0.25 g ET, Tid)	12 weeks
Feng (2019)	RCT*	90 (45/45)	(25/20)/(26/19)	53-80(Mean 64.2 ± 7.5)/54-81(Mean 64.7 ± 7.3)	Mean 4.48 ± 1.59/4.55 ± 1.67	HLJD	M (0.25 g ET, Tid)	12 weeks
Xing et al. (2017)	RCT	106 (51/55)	(27/24)/(28/27)	35-61(Mean 58.3 ± 12.6)/38-62(Mean 56.6 ± 11.7)	Mean 3.5 ± 1.8/3.6 ± 1.7	HLJD	M (0.5 g ET, Tid)	2 weeks
Yang and Wang. (2013)	RCT	66 (33/33)	(22/11)/(20/13)	25-65 (Mean 42 ± 16/40 ± 15)	Unknown	HLJD	M	24 weeks
Zhang (2014)	RCT	260 (130/130)	140/120	42 -65 (Mean 51.9 ± 5.8)	Unknown	HLJD	M (0.25 g ET, Tid)	12 weeks
Chen and Wang. (2021)	RCT*	99 (50/49)	(28/22)/(25/24)	38-70(Mean54.37 ± 2.56)/39-71(Mean54.41 ± 2.24)	Mean10.27 ± 3.96/10.25 ± 3.94	HLJD	M (0.5 g ET, Tid)	12 weeks
Wei et al. (2021)	RCT	60 (30/30)	Unknown	40-65(Mean53.68 ± 5.02)/39-68(Mean55.05 ± 4.82)	Mean 5.1 ± 0.5/5.00 ± 1.01	HLJD	M (0.5 g ET, Tid)	Unknown
Song et al. (2022)	RCT*	100 (50/50)	(34/16)/(32/18)	29-70(Mean 52.45 ± 7.12)/30-70(Mean 52.48 ± 7.15)	4 weeks-6 years/3 weeks-6 years	HLJD	M (0.25 g ET, Tid)	8 weeks
Chen (2018)	RCT	60 (30/30)	(12/18)/(12/18)	Mean 58.57/58.9	Unknown	HLWD	M (0.5 g ET, Tid)	8 weeks
Dong (2017)	RCT*	70 (35/35)	(20/15)/(19/16)	40-65 (Mean 51.3 ± 5.1/52.8 ± 4.7)	Unknown	HLWD	M (0.5 g ET, Tid)	12 weeks
Ji (2017)	RCT	60 (30/30)	(14/16)/(12/18)	20-79	Mean 4.12 ± 3.45/4.66 ± 2.87	HLWD	M (0.5 g ET, Tid)	12 weeks
Zhang (2019)	RCT	60 (30/30)	(17/13)/(13/17)	30-65 (Mean47.5 ± 7.7/48.53 ± 8.59)	Mean 3.64 ± 2.63/3.78 ± 3.42	HLWD	M (0.5 g ET, Tid)	12 weeks
Fu (2021)	RCT*	120 (60/60)	(30/30)/(31/29)	20-70(Mean55.72 ± 1.62)/19-71(Mean56.59 ± 1.71)	Mean 7.21 ± 2.62/7.35 ± 2.23	HLWD	M (0.85 g ET, Tid)	
Liu et al. (2021)	RCT*	68 (34/34)	(19/15)/(11/23)	Mean 55 ± 11/55 ± 7	Unknown	HLWD	M (0.5 g ET, Tid)	8 weeks
Pan et al. (2021)	RCT	80 (41/39)	(20/21)/(18/21)	40-60(Mean 50.1 ± 5.5)/42-62(Mean51.2 ± 5.4))	Mean (4.32 ± 0.19/4.12 ± 0.23	HLWD	M (0.5 g once a day)	16 weeks
Wang et al. (2021)	RCT*	60 (30/30)	(15/15)/(13/17)	Mean 60.07 ± 7.1/58.70 ± 6.97	Unknown	HLWD	M (0.5 g ET, Tid)	8 weeks
Wang Y 2022	RCT*	50 (25/25)	(16/9)/(15/10)	50-70	1-6	HLWD	M (0.5 g once a day)	16
Zhang (2022)	RCT	76 (38/38)	(18/20)/(19/19)	Mean 69.42 ± 12.4/68.92 ± 11.89	1-6 (month)	HLWD	M (0.25 g ET, Bid)	8

Notes: E, experimental group; C, control group; M, metformin; *, Random number table method; #, Double-blind; Qd, One time a day; Bid, Two times a day; Tid, Three times a day; ET, each time.

**TABLE 2:** Details of the HLPs for each study.

Interventions	References	Prescription
DHHL decoction	Li et al. (2017)	*Coptis chinensis* Franch. 5 g, *Rheum palmatum* L. 10 g, Scutellaria baicalensis Georgi 5 g
	Wu et al. (2019)	*Coptis chinensis* Franch. 3 g, *Rheum palmatum* L. 6 g, Scutellaria baicalensis Georgi 10 g
	Zou and lao, (2016)	*Coptis chinensis* Franch. 5 g, *Rheum palmatum* L. 10 g, Scutellaria baicalensis Georgi 5 g
GGQL decoction	Fan et al. (2017)	*Coptis chinensis* Franch. 10 g, *Scutellaria baicalensis* Georg 15 g, *Pueraria lobata* (Willd.) Ohwi 30 g, *Glycyrrhiza uralensis* Fisch. 6 g
	Fu, (2017)	*Coptis chinensis* Franch. 30 g, *Scutellaria baicalensis* Georg 20 g, *Pueraria lobata* (Willd.) Ohwi 50 g, *Glycyrrhiza uralensis* Fisch. 6 g
	Jin et al. (2019)	*Coptis chinensis* Franch. 10 g, *Scutellaria baicalensis* Georg 15 g, *Pueraria lobata* (Willd.) Ohwi 30 g, *Glycyrrhiza uralensis* Fisch. 6 g
	Pang et al. (2018)	*Coptis chinensis* Franch. 22.5 g, *Scutellaria baicalensis* Georg 22.5 g, *Pueraria lobata* (Willd.) Ohwi 60 g, *Glycyrrhiza uralensis* Fisch. 15 g, *Zingiber officinale* Roscoe 3.5 g
	Xiong, (2019)	*Coptis chinensis* Franch. 22.5 g, *Scutellaria baicalensis* Georg 22.5 g, *Pueraria lobata* (Willd.) Ohwi 60 g, *Glycyrrhiza uralensis* Fisch. 15 g, *Zingiber officinale* Roscoe 3.5 g
	Zhang et al. (2019)	*Coptis chinensis* Franch. 5 g, *Scutellaria baicalensis* Georg 20 g, *Pueraria lobata* (Willd.) Ohwi 20 g, *Glycyrrhiza uralensis* Fisch. 5 g
	Zhang et al. (2018)	*Coptis chinensis* Franch. 20 g, *Scutellaria baicalensis* Georg 20 g, *Pueraria lobata* (Willd.) Ohwi 30 g, *Glycyrrhiza uralensis* Fisch. 9 g
HLEJ decoction	Liu, 2006	*Coptis chinensis* Franch. 10 g, *Scutellaria baicalensis* Georgi 15 g, *Paeonia anomala* L*.* 15 g, Asparagus acutifolius L 20 g, Colla corii asini 15 g
	Liu, (2017)	*Coptis chinensis* Franch. 10 g, *Scutellaria baicalensis* Georgi 15 g, *Paeonia anomala* L*.* 15 g, Asparagus acutifolius L 20 g, Colla corii asini 15 g
	Gao, (2020)	*Coptis chinensis* Franch. 8 g, *Rheum palmatum* L. 10 g, *Paeonia anomala* L*.* 15 g, Colla corii asini 10 g, Semen Ziziphi Spinosae 25 g, *Rehmannia glutinosa* (Gaertn.) DC. 20 g, *Polygonum multiflorum* Thunb. 15 g, *Anemarrhena asphodeloides* Bunge 10 g, fresh egg yolk 1
	Wang, (2020)	*Coptis chinensis* Franch. 10 g, Scutellaria baicalensis Georgi 15 g, *Paeonia anomala* L*.* 15 g, Asparagus acutifolius L 20 g, Colla corii asini 15 g)
	Zhou et al. (2022)	*Coptis chinensis* Franch. 10 g, Scutellaria baicalensis Georgi 6 g, *Paeonia anomala* L*.* 10 g, fresh egg yolk 1, Colla corii asini 10 g
HLJD decoction	Ding, (2018)	*Coptis chinensis* Franch. 12 g, *Scutellaria baicalensis* Georgi 9 g, *Phellodendron amurense* Rupr. 9 g, *Gardenia jasminoides* J.Ellis 12 g
	Feng, (2019)	*Coptis chinensis* Franch. 12 g, *Scutellaria baicalensis* Georgi 9 g, *Phellodendron amurense* Rupr. 9 g, *Gardenia jasminoides* J.Ellis 12 g
	Xing et al. (2017)	*Coptis chinensis* Franch. 12 g, *Scutellaria baicalensis* Georgi 12 g, *Phellodendron amurense* Rupr. 9 g, *Gardenia jasminoides* J.Ellis 12 g
	Yang and Wang, (2013)	*Coptis chinensis* Franch. 15 g, *Scutellaria baicalensis* Georgi 10 g, *Phellodendron amurense* Rupr. 6 g, *Gardenia jasminoides* J.Ellis 10 g
	Zhang, (2014)	*Coptis chinensis* Franch. 9 g, *Scutellaria baicalensis* Georgi 6 g, *Phellodendron amurense* Rupr. 6 g, *Gardenia jasminoides* J.Ellis 9 g
	Chen and Wang, (2021)	*Coptis chinensis* Franch. 10 g, *Scutellaria baicalensis* Georgi 10 g, *Phellodendron amurense* Rupr. 10 g, *Gardenia jasminoides* J.Ellis 10 g, *Ophiopogon japonicus* (Thunb.) Ker Gawl. 12 g, *Scrophularia ningpoensis* Hemsl. 12 g, *Rehmannia glutinosa* (Gaertn.) DC. 12 g, *Forsythia suspensa* (Thunb.) Vahl 15 g, *Taraxacum mongolicum* Hand.-Mazz. 15 g, *Lonicera japonica* Thunb. 20 g
	Wei et al. (2021)	*Coptis chinensis* Franch. 10 g, *Scutellaria baicalensis* Georgi 15 g, *Phellodendron amurense* Rupr. 15 g, *Gardenia jasminoides* J.Ellis 10 g
	Song et al., 2022	*Coptis chinensis* Franch. 12 g, *Scutellaria baicalensis* Georgi 12 g, *Phellodendron amurense* Rupr. 9 g, *Gardenia jasminoides* J.Ellis 12 g
HlLWD decoction	Chen, (2018)	*Coptis chinensis* Franch. 9 g, *Scutellaria baicalensis* Georgi 9 g, *Pueraria lobata* (Willd.) Ohw 30 g, *Trichosanthes kirilowii Maxim.* 30 g, *Citrus reticulata* Blanco 15 g, *Pinellia ternata* (Thunb.) Makino 9 g, *Bambusa tuldoides* Munro 9 g, *Curcuma phaeocaulis* Valeton 9 g, *Fritillaria thunbergii* Miq. 15 g, *Poria Cocos* (Schw.) Wolf. 15 g, *Atractylodes macrocephala* Koidz. 15 g, *Salvia miltiorrhiza* Bunge 30 g, *Bupleurum chinense* DC*.* 15 g
	Dong, (2017)	*Coptis chinensis* Franch. 9 g, *Citrus reticulata* Blanco 12 g, *Pinellia ternata* (Thunb.) Makino 9 g, *Bambusa tuldoides* Munro 6 g, *Poria Cocos* (Schw.) Wolf. 15 g, *Atractylodes macrocephala* Koidz. 15 g, *Salvia miltiorrhiza* Bunge 15 g, *Citrus aurantium L.* 12 g, *Astragalus propinquus* Schischkin 20 g, *Glycyrrhiza uralensis* Fisch. 6 g, Trigonellafoenum-graeeum 15 g
	Ji, (2017)	*Coptis chinensis* Franch. 9 g, *Scutellaria baicalensis* Georgi 12 g, *Trichosanthes kirilowii Maxim* 30 g, *Citrus reticulata* Blanco 15 g, *Pinellia ternata* (Thunb.) Makino 9 g, *Poria Cocos* (Schw.) Wolf. 15 g, *Atractylodes macrocephala* Koidz. 15 g, *Pueraria lobata* (Willd.) Ohw 15 g, *Salvia miltiorrhiza* Bunge 30 g, *Citrus aurantium L.* 6 g, *Rheum palmatum* L. 6 g
	Zhang, (2019)	*Coptis chinensis* Franch. 6 g, *Citrus reticulata* Blanco 10 g, *Citrus reticulata* Blanco 10 g, *Pinellia ternata* (Thunb.) Makino 6 g, *Poria Cocos* (Schw.) Wolf. 15 g, *Atractylodes macrocephala* Koidz. 15 g, *Pueraria lobata* (Willd.) Ohw 15 g, *Salvia miltiorrhiza* Bunge 15 g, *Agastache rugosa* (Fisch. and C.A.Mey.) Kuntze 10 g, *Magnolia officinalis* Rehder and E.H.Wilson 6 g, *Coix lacryma-jobi* L. 15 g, *Glycyrrhiza uralensis* Fisch*.* 3 g
	Fu, (2021)	*Coptis chinensis* Franch. 10 g, *Poria Cocos* (Schw.) Wolf. 20 g, *Citrus aurantium L.* 10 g, *Pinellia ternata* (Thunb.) Makino 15 g, *Bambusa tuldoides* Munro 10 g, *Citrus reticulata* Blanco 15 g, *Pueraria lobata* (Willd.) Ohw 15 g, *Eupatorium fortunei* Turcz. 10 g, *Glycyrrhiza uralensis* Fisch. 10 g
	Liu et al. (2021)	*Coptis chinensis* Franch. 10 g, *Poria Cocos* (Schw.) Wolf. 20 g, *Citrus aurantium L.* 10 g, *Pinellia ternata* (Thunb.) Makino 15 g, *Bambusa tuldoides* Munro 10 g, *Citrus reticulata* Blanco 15 g, *Pueraria lobata* (Willd.) Ohw 15 g, *Eupatorium fortunei* Turcz. 10 g, *Glycyrrhiza uralensis* Fisch. 10 g
	Pan et al. (2021)	*Coptis chinensis* Franch. 15 g, *Poria Cocos* (Schw.) Wolf. 15 g, *Citrus aurantium L.* 12 g, *Pinellia ternata* (Thunb.) Makino 6 g, *Bambusa tuldoides* Munro 15 g, *Citrus reticulata* Blanco 12 g, *Glycyrrhiza uralensis* Fisch. 15 g, *Zingiber officinale* Roscoe 10 g, *Atractylodes macrocephala* Koidz. 15 g
	Wang et al. (2021)	*Coptis chinensis* Franch. 15 g, *Poria Cocos* (Schw.) Wolf. 15 g, *Citrus aurantium L.* 12 g, *Pinellia ternata* (Thunb.) Makino 6 g, *Bambusa tuldoides* Munro 15 g, *Citrus reticulata* Blanco 12 g, *Glycyrrhiza uralensis* Fisch. 15 g, *Zingiber officinale* Roscoe 10 g, *Atractylodes macrocephala* Koidz. 15 g
	Wang, (2020)	*Coptis chinensis* Franch. 15 g, *Poria Cocos* (Schw.) Wolf. 15 g, *Citrus aurantium L.* 12 g, *Pinellia ternata* (Thunb.) Makino 6 g, *Bambusa tuldoides* Munro 15 g, *Citrus reticulata* Blanco 12 g, *Glycyrrhiza uralensis* Fisch. 15 g, *Zingiber officinale* Roscoe 10 g, *Atractylodes macrocephala* Koidz. 15 g
	Zhang, 2022	*Coptis chinensis* Franch. 15 g, *Poria Cocos* (Schw.) Wolf. 15 g, *Citrus aurantium L.* 12 g, *Pinellia ternata* (Thunb.) Makino 6 g, *Bambusa tuldoides* Munro 15 g, *Citrus reticulata* Blanco 12 g, *Glycyrrhiza uralensis* Fisch. 15 g, *Zingiber officinale* Roscoe 10 g, *Atractylodes macrocephala* Koidz. 15 g

### 3.3 Quality assessment

**FIGURE 2:**
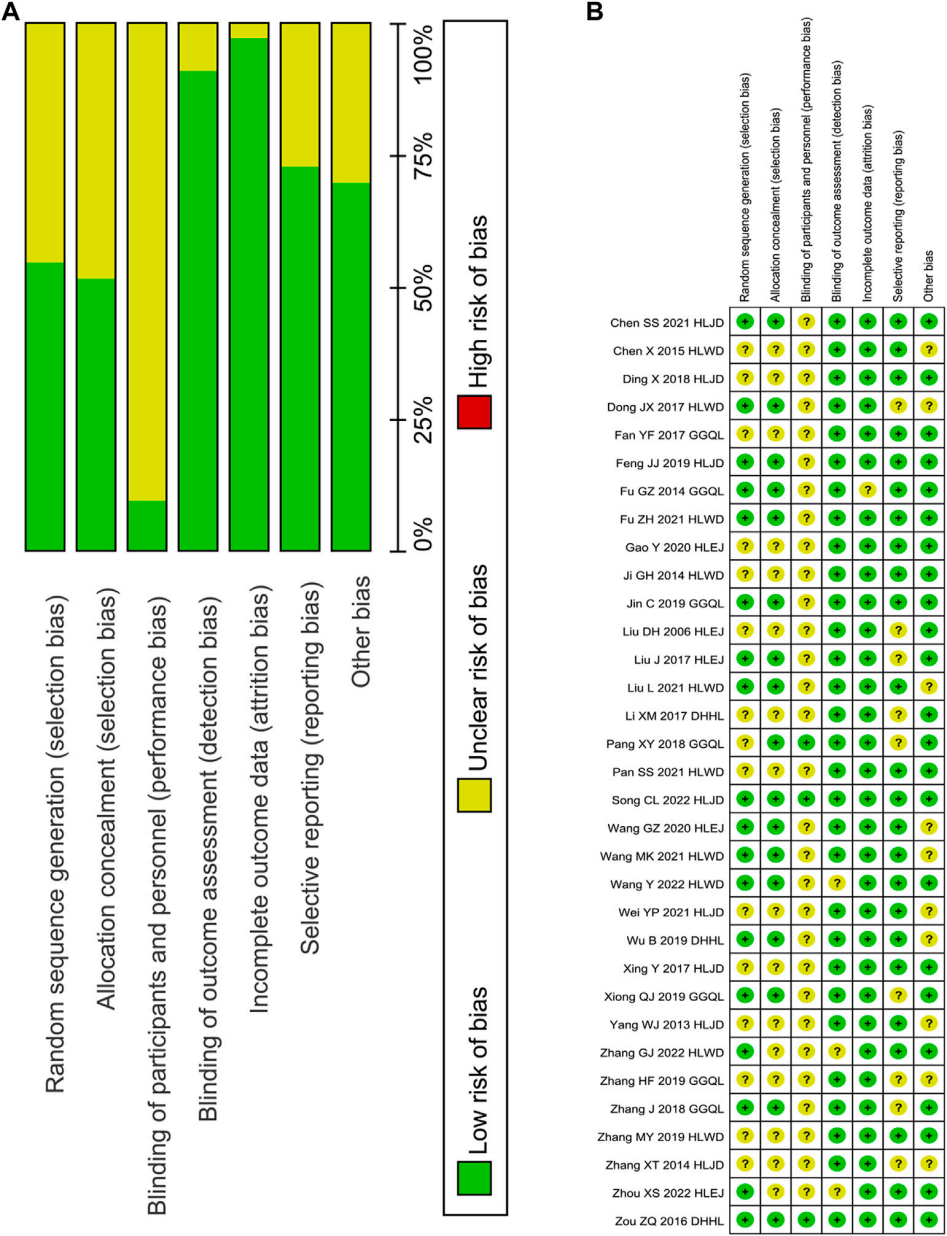
Risk of bias graph. Note: **(A)**, judgements about each risk of bias item presented as percentages across all included studies; **(B)**, judgements about each risk of bias item for each included study.

A total of 33 RCTs were identified in this study (Figure 2), of which 16 used the random number table method to generate random sequences (Chen et al., 2012; Zou and Lao, 2016; Dong, 2017; Fu, 2017; Liu, 2017; Zhang et al., 2018; Feng, 2019; Jin et al., 2019; Wu et al., 2019; Xiong, 2019; Wang 2020; Wang, 2020; Fu, 2021; Liu et al., 2021; Song et al., 2022; Wang et al., 2021), and others only mentioned randomly assigned participants. Three trials used the double-blind method for participants and personnel (Zou and Lao, 2016; Pang et al., 2018; Song et al., 2022), and others provided no detailed information. The risk of detection bias was low in all trials, because FBG, 2hPG, HbA1c, FINS, HOMA-IR, TC, TG, LDL-c, HDL-c, LDL-c, and GD levels were evaluated based on objective criteria. In the study conducted by Fu (2017), three patients in the experimental group and the control group withdrew from the trial (9% exit rate). The remaining trials without the loss of follow-up patients or with the loss of follow-up rate <5% were described as having a low-attrition bias. For the reporting bias, nine trials with only positive results were determined as unclear (Zhang, 2014; Dong, 2017; Li et al., 2017; Liu, 2006; Liu, 2017; Pang et al., 2018; Zhang et al., 2018; Xiong, 2019; Zhang, 2019). For other bias, ten trials were unclear in the sex of the patient, course of the disease, and course of treatment (Chen et al., 2012; Dong, 2017; Liu et al., 2021; Wang, 2020; Wang et al., 2021; Wei et al., 2021; Wu et al., 2019; Yang and Wang, 2013; Zhang, 2014; Zhang, 2019). Meanwhile, others with detailed information presented a low risk.

**FIGURE 3:**
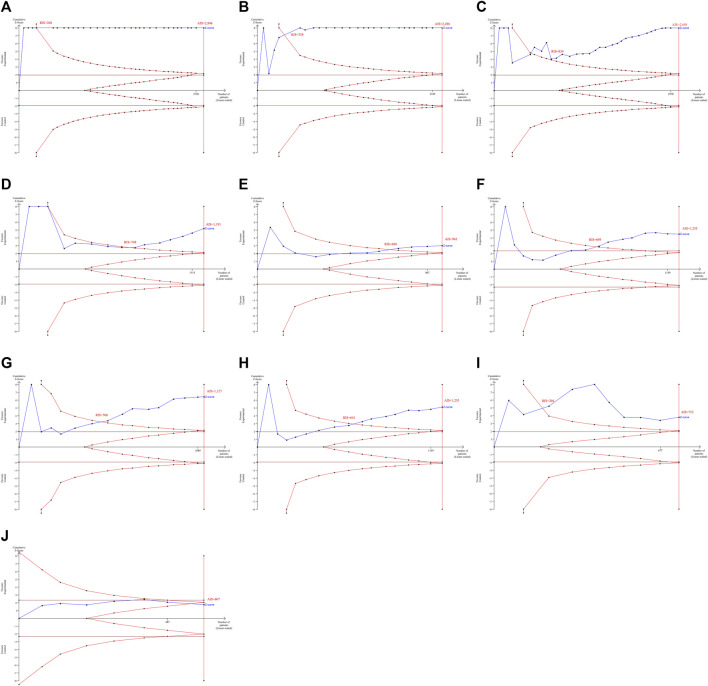
TSA analysis for the effectiveness of HLPs in improving T2DM. Note: **(A)** Represents FBG; **(B)** Represents 2hPG; **(C)** Represents HbA1c; **(D)** Represents FINS; **(E)** Represents HOMA-IR; **(F)** Represents TC; **(G)** Represents TG; **(H)** Represents LDL-c; **(I)** Represents HDL-c; **(J)** Represents GD.

TSA results revealed that the AIS exceeded the RIS for the effectiveness of HLPs in improving FBG (AIS 2,846 was larger than RIS 268), 2hPG (AIS 2,280 was larger than RIS 338), HbA1c (AIS 2,698 was larger than RIS 834), FINS (AIS 1,191 was larger than RIS 748), HOMA-IR (AIS 943 was larger than RIS 680), TC (AIS 1,235 was larger than RIS 695), TG (AIS 1,127 was larger than RIS 506), LDL-c (AIS 1,235 was larger than RIS 641), and HDL-c (AIS 753 was larger than RIS 204), and their cumulative Z-curves crossed the trial sequential monitoring boundary (Figures 3A–I), indicating that their current evidence was sufficient to draw a reliable conclusion. However, the AIS didn’t exceed the RIS for the effectiveness of HLPs in improving GD (Figure 3J), indicating that the current evidence was’t sufficient to draw a reliable conclusion. GRADE assessment suggested that the quality of evidence was moderate for the effectiveness of HLPs in improving FBG, but the quality of evidence was low for 2hPG, HbA1c, FINS, HOMA-IR, TC, TG, LDL-c, and HDL-c, even very low for GD (Table 3).

**TABLE 3:** GRADE evidence profile of clinical efficacy.

Quality assessment					Effect	Quality	Importance
Risk of bias	Inconsistency	Indirectness	Imprecision	Other considerations			
FBG							
Serious^a^	No serious inconsistency	No serious indirectness	No serious imprecision	None	MD 1.16 lower (1.24–1.07 lower)	ÅÅÅO MODERATE	CRITICAL
2hPG							
Serious^b^	Serious^b^	No serious indirectness	No serious imprecision	None^c^	MD 1.64 lower (1.84–1.43 lower)	ÅÅOO LOW	CRITICAL
HbA1c							
Serious^a^	Serious^b^	No serious indirectness	No serious imprecision	None^c^	MD 0.78 lower (0.96–0.60 lower)	ÅÅOO LOW	CRITICAL
FINS							
Serious^a^	Serious^b^	No serious indirectness	No serious imprecision	None	MD 1.94 lower (2.68–1.2 lower)	ÅÅOO LOW	CRITICAL
HOMA-IR							
Serious^a^	Serious^b^	No serious indirectness	No serious imprecision	None	MD 0.77 lower (1.28–0.27 lower)	ÅÅOO LOW	CRITICAL
TC							
Serious^a^	Serious^b^	No serious indirectness	No serious imprecision	None	MD 0.70 lower (1.00–0.39 lower)	ÅÅOO LOW	IMPORTANT
TG							
Serious^a^	Serious^b^	No serious indirectness	No serious imprecision	None	MD 0.59 lower (0.76–0.41 lower)	ÅÅOO LOW	IMPORTANT
HDL-c							
Serious^a^	Serious^b^	No serious indirectness	No serious imprecision	None	MD 0.21 lower (0.32–0.1 lower)	ÅÅOO LOW	IMPORTANT
LDL-c							
Serious^a^	Serious^b^	No serious indirectness	No serious imprecision	None	MD 0.70 lower (0.97–0.43 lower)	ÅÅOO LOW	IMPORTANT
GD							
Serious^a^	Serious^b^	No serious indirectness	No serious imprecision	Reporting bias^c^	32 fewer per 1,000	ÅOOO VERY LOW	IMPORTANT
					31 fewer per 1,000		

a Note: Most domain had unclear methodological bias risk.

b The trials included had obvious heterogeneity.

c The number of included studies is insufficient.

### 3.4 Effectiveness of HLPs for T2DM

#### 3.4.1 HLPs for FBG

**FIGURE 4:**
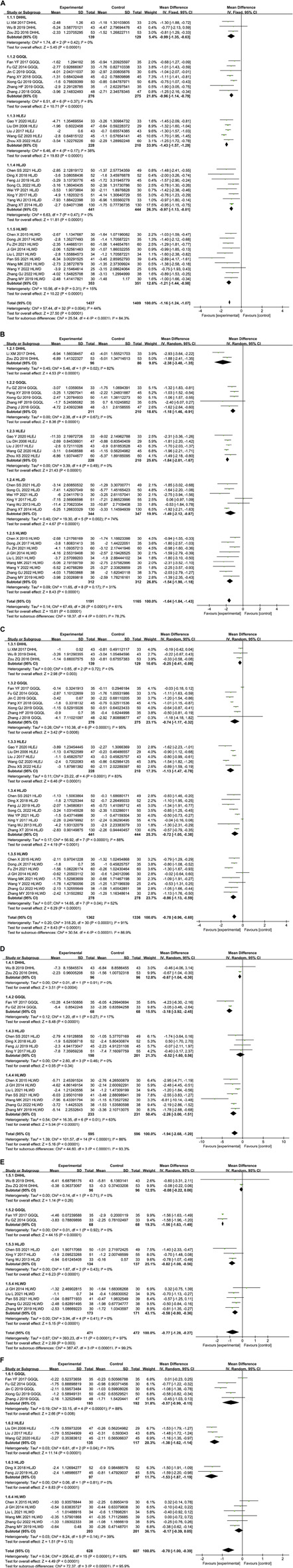
(Continued).

As shown in Figure 4A, a total of 33 trials comprising 1,437 subjects in the experimental group and 1,409 subjects in the control group evaluated the effectiveness of HLPs in improving FBG. Subgroups were divided depending on the type of HLPs for FBG. The results indicated that T2DM patients who received metformin in combination with DHHL decoction (MD = −0.99%, 95% CI: −1.35 to −0.63, and *p* < 0.00001), GGQL decoction (MD = −0.96%, 95% CI: −1.14 to −0.79, and *p* < 0.00001), HLEJ decoction (MD = −1.43%, 95% CI: −1.57 to −1.29, and *p* < 0.00001), HLJD decoction (MD = −0.97%, 95% CI: −1.13 to −0.81, and *p* < 0.00001), and HLWD decoction (MD = −1.21%, 95% CI: −1.44 to −0.98, and *p* < 0.00001) respectively were more likely to have reduced FBG relative to those with metformin alone. No significant heterogeneity was indicated in DHHL decoction (I^2^ = 0%), GGQL decoction (I^2^ = 8%), HLEJ decoction (I^2^ = 38%), HLJD decoction (I^2^ = 0%), and HLWD decoction (I^2^ = 15%) for FBG. Overall analysis showed that compared with metformin alone, HLPs combined with metformin improved FBG more (I^2^ = 44%, MD = −1.16%, 95% CI: −1.24 to −1.07, and *p* < 0.00001).

#### 3.4.2 HLPs for 2hPG

As shown in Figures 4A,B total of 27 trials comprising 1,191 subjects in the experimental group and 1,165 subjects in the control group compared the 2hPG among patients with T2DM. Subgroup analysis was performed based on the type of HLPs for 2hPG. Patients who received the following decoctions, combined with metformin, were more likely to exhibit reduced 2hPG relative to the controls: DHHL decoction (MD = −2.38, 95% CI: −3.40 to −1.35, and *p* < 0.00001), GGQL decoction (MD = −1.18%, 95% CI: −1.46 to −0.91, and *p* < 0.00001), HLEJ decoction (MD = −1.84%, 95% CI: −2.01 to −1.67, and *p* < 0.00001), HLJD decoction (MD = −1.49%, 95% CI: −2.12 to −0.87, and *p* < 0.00001), and HLWD decoction (MD = −1.54%, 95% CI: −1.90 to −1.18, and *p* < 0.00001). No significant heterogeneity was found in the following: GGQL decoction (0%), HLEJ decoction (I^2^ = 0%), and HLWD decoction (I^2^ = 31%) for 2hPG. By contrast, significant heterogeneity was found in DHHL decoction (I^2^ = 82%) and HLJD decoction (I^2^ = 74%) for 2hPG. Overall analysis indicated that decreases in 2hPG were greater in groups treated using HLPs combined with metformin (I^2^ = 61%, MD = −1.64%, 95% CI: −1.84 to −1.43, and *p* < 0.00001).

#### 3.4.3 HLPs for HbA1c

A total of 31 trials comprising 1,362 subjects in the experimental group and 1,336 subjects in the control group assessed changes in HbA1c levels (Figure 4C). Subgroup analysis was used in different types of HLPs for HbA1c. Patients who received the following decoctions in combination with metformin were more likely to exhibit reduced HbA1c relative to that with metformin alone: DHHL decoction (MD = −0.25%, 95% CI: −0.41 to −0.09, and *p* = 0.003), GGQL decoction (MD = −0.74%, 95% CI: −1.17 to −0.32, and *p* = 0.0006), HLEJ decoction (MD = −1.13%, 95% CI: −1.47 to −0.78, and *p* < 0.00001), HLJD decoction (MD = −0.72%, 95% CI: −1.05 to −0.38, and *p* < 0.00001), and HLWD decoction (MD = −0.86%, 95% CI: −1.13 to −0.59, and *p* < 0.00001). No significant heterogeneity in DHHL decoction for HbA1c was found (I^2^ = 0%). However, GGQL decoction (I^2^ = 95%), HLEJ decoction (I^2^ = 83%), HLJD decoction (I^2^ = 88%), and HLWD decoction (I^2^ = 52%) for HbA1c exhibited significant heterogeneity. Overall analysis indicated that HLPs combined metformin provided additional benefits to reduce HbA1c (I^2^ = 91%, MD = −0.78%, 95% CI:−0.96 to −0.60, and *p* < 0.00001).

#### 3.4.4 HLPs for FINS

A total of 15 trials comprising 595 subjects in the experimental group and 596 subjects in the control group assessed changes in FINS levels (Figure 4D). Subgroup analysis was used in different types of HLPs for FINS. Patients who received the following decoctions in combination with metformin were more likely to exhibit reduced FINS relative to that with metformin alone: DHHL decoction (MD = −0.67%, 95% CI: −1.04 to −0.30, and *p* = 0.0004), GGQL decoction (MD = −3%.18%, 95% CI: −3.92 to −2.45, and *p* < 0.00001), and HLWD decoction (MD = −2.26%, 95% CI:−3.00 to −1.51, and *p* < 0.00001). No significant heterogeneity in DHHL decoction (I^2^ = 0%) and GGQL decoction (I^2^ = 17%) for FINS was found, while HLWD decoction for FINS had significant heterogeneity (I^2^ = 63%). In addition, HLJD decoction for FINS was not statistically significant (MD = -0.52, 95% CI: -1.60 to 0.56, and *p* = 0.34). Overall analysis indicated that patients treated with HLPs in combination with metformin were more likely to reduce FINS (I^2^ = 86%, MD = −1.94%, 95% CI: −2.68 to −1.20, and *p* < 0.00001).

#### 3.4.5 HLPs for HOMA-IR

A total of 12 trials comprising 471 subjects in the experimental group and 472 subjects in the control group reported HOMA-IR as an outcome (Figure 4E). Subgroup analysis was used in different types of HLPs for HOMA-IR. The results showed that patients who received metformin in combination with GGQL decoction (MD = −1.56%, 95% CI: −1.63 to −1.49, and *p* < 0.00001), HLJD decoction (MD = −0.82%, 95% CI: −1.08 to −0.56, and *p* < 0.00001), and HLWD decoction (MD = −0.58%, 95% CI: −0.80 to −0.36, and *p* < 0.00001) respectively were more likely to have reduced HOMA-IR relative to those with metformin alone. No significant heterogeneity was indicated in GGQL decoction (I^2^ = 0%), HLJD decoction (I^2^ = 0%), and HLWD decoction (I^2^ = 0%) for HOMA-IR. However, DHHL decoction for HOMA-IR was not statistically significant (MD = −0.08%, 95% CI: −0.22 to 0.06, and *p* = 0.26). Overall analysis showed that HLPs combined with metformin were more likely to reduce HOMA-IR compared with metformin alone (I^2^ = 97%, MD = −0.77%, 95% CI: −1.28 to −0.27, and *p* = 0.003).

#### 3.4.6 HLPs for blood lipids

A total of 16 trials comprising 628 subjects in the experimental group and 607 subjects in the control group evaluated the effectiveness of HLPs in improving TC (Figure 4F). Subgroups were divided depending on the type of HLPs for TC. The results revealed that patients who received metformin in combination with GGQLdecoction (MD = −0.57%, 95% CI:−0.99 to −0.15, and *p* = 0.008), HLEJ decoction (MD = −1.38%, 95% CI:−1.62 to −1.14, and *p* < 0.00001), and HLJD decoction (MD = −1.53%, 95% CI:−1.87 to −1.19, and *p* < 0.00001) respectively were more likely to have reduced TC relative to those with metformin alone. No significant heterogeneity was indicated in HLJD decoction for TC (I^2^ = 0%), while significant heterogeneity was found in GGQL decoction (I^2^ = 88%) and HLEJ decoction (I^2^ = 70%) for TC. In addition, HLWD decoction for TC was not statistically significant (MD = −0.17%, 95% CI: −0.39 to 0.05, and *p* = 0.13). Overall analysis indicated that decreases in TC were greater in groups treated using HLPs combined with metformin (I^2^ = 93%, MD = −0.70%, 95% CI: −1.00 to −0.39, and *p* < 0.00001).

A total of 15 trials comprising 593 subjects in the experimental group and 572 subjects in the control group evaluated the curative effect of HLPs in improving TG (Figure 4G). Subgroups were divided depending on the type of HLPs for TG. Patients who received the following decoctions, combined with metformin, were more likely to exhibit reduced TG relative to the controls: GGQL decoction (MD = −0.46%, 95% CI:−0.78 to −0.13, and *p* = 0.006), HLEJ decoction (MD = -1.19%, 95% CI:−1.84 to −0.55, and *p* = 0.0003), HLJD decoction (MD = −0.48%, 95% CI:−0.58 to −0.38, and *p* < 0.00001), and HLWD decoction (MD = −0.32%, 95% CI:−0.49 to −0.14, and *p* = 0.0003). No significant heterogeneity was found in HLJD decoction (I^2^ = 0%) and HLWD decoction (I^2^ = 18%) for TG, while significant heterogeneity was found in GGQL decoction (I^2^ = 89%) and HLEJ decoction (I^2^ = 96%) for TG. Overall analysis indicated that decreases in TG were greater in groups treated using HLPs combined with metformin (I^2^ = 89%, MD = −0.57%, 95% CI: −0.74 to −0.40, and *p* < 0.00001).

A total of 16 trials comprising 628 subjects in the experimental group and 607 subjects in the control group evaluated the curative effect of HLPs in improving LDL-c (Figure 4H). Subgroups were divided depending on the type of HLPs for LDL-c. Patients who received the following decoctions, combined with metformin, were more likely to exhibit reduced LDL-c relative to the controls: GGQLdecoction (MD = −0.41%, 95% CI:−0.54 to −0.29, and *p* < 0.00001), HLEJ decoction (MD = −1.17%, 95% CI:−1.77 to −0.57, and *p* = 0.0001), and HLJD decoction (MD = −1.62%, 95% CI:−1.85 to −1.40, and *p* < 0.00001). No significant heterogeneity was found in GGQL decoction (I^2^ = 11%) and HLJD decoction (I^2^ = 0%) for LDL-c, while significant heterogeneity was found in HLEJ decoction for LDL-c (I^2^ = 94%). In addition, HLWD decoction for LDL-c was not statistically significant (MD = −0.36%, 95% CI: −0.71 to 0.00, and *p* = 0.05). Overall analysis indicated that HLPs combined metformin provided additional benefits to reduce LDL-c (I^2^ = 94%, MD = −0.70%, 95% CI: −0.97 to −0.43, and *p* < 0.00001).

A total of 10 trials comprising 378 subjects in the experimental group and 375 subjects in the control group evaluated the curative effect of HLPs in improving HDL-c (Figure 4I). Subgroups were divided depending on the type of HLPs for HDL-c. The results revealed that patients who received metformin in combination with GGQL decoction (MD = −0.32%, 95% CI:−0.54 to −0.10, and *p* = 0.005) and HLJD decoction (MD = −0.32%, 95% CI:−0.41 to −0.23, and *p* < 0.00001) respectively were more likely to have reduced HDL-c relative to those with metformin alone. No significant heterogeneity was indicated in HLJD decoction for HDL-c (I^2^ = 0%), while significant heterogeneity was found in GGQL decoction for HDL-c (I^2^ = 81%). In addition, HLWD decoction for HDL-c was not statistically significant (MD = −0.07%, 95% CI: −0.17 to 0.03, and *p* = 0.19). Overall analysis showed that compared with metformin alone, HLPs combined with metformin improved HDL-c more (I^2^ = 84%, MD = −0.21%, 95% CI: −0.32 to −0.10, and *p* = 0.0002).

#### 3.4.7 HLPs for GD

A total of seven trials comprising 313 subjects in the experimental group and 294 subjects in the control group conducted analysis of HLPs for GD (Figure 4J). Patients who received HLPs can’t reduce GD relative to those with metformin alone (OR = 0.54%, 95% CI:0.26 to 1.10, and *p* = 0.09).

### 3.5 Sensitivity analysis

The results in Table 4 suggest that patients with T2DM in the experimental group show improved FBG, 2hPG, HbA1c, FINS, HOMA-IR, TC, TG, LDL-c, and HDL-c relative to those in the control group. However, changes in the effectiveness of HLPs in improving 2hPG, HbA1c, FINS, HOMA-IR, TC, TG, LDL-c, and HDL-c showed significant heterogeneity. With regard to the subgroup sensitivity analysis, after excluding some underestimated or overestimated trials, the heterogeneity of the majority of studies was significantly reduced, including the following: HLJD for 2hPG, HLWD for FINS; GGQL, HLEJ, HLJD, and HLWD for HbA1c; GGQL and HLEJ for TC; GGQL and HLEJ for TG; HLEJ and HLWD for LDL-c; and DHHL for HDL-c. However, no statistically significant difference was found in DHHL for 2hPG, HLJD for FINS, and DHHL for HOMA-IR.

**TABLE 4:** Sensitivity analysis via excluding the under or over estimated trials.

Analysis	MD (95% CI)	I2 (%)	*p* (Z test)	Excluded studies [reference]	MD (95% CI)	I^2^ (%)	*p* (Z test)
2hPG-DHHL	−2.38 [−3.40,−1.35]	82%	*p* < 0.00001		Not applicable		
2hPG-HLJD	−1.49 [−2.12,−0.87]	74%	*p* < 0.00001	Xing et al. (2017)	−1.80 [−2.23, −1.37]	42	*p* < 0.00001
HbA1c-GGQL	−0.74 [−1.17,−0.32]	95%	*p* = 0.0006	Fan et al. (2017)	−1.04 [−1.16,−0.92]	0	*p* < 0.00001
				Xiong, (2019)			
				Zhang, (2019)			
HbA1c-HLEJ	−1.13 [−1.47, −0.78]	83%	*p* < 0.00001	Gao, (2020)	−0.85 [−0.99, −0.70]	0	*p* < 0.00001
				Wang, (2020)			
HbA1c-HLJD	−0.72 [−1.05, −0.38]	88%	*p* < 0.0001	Feng, (2019)	−0.64 [−0.81, −0.48]	15	*p* < 0.00001
				Xing et al. (2017)			
HbA1c-HLWD	−0.86 [−1.13, −0.59]	52%	*p* < 0.00001	Ji, (2017)	−0.96 [−1.15, −0.77]	8	*p* < 0.00001
FINS-HLJD	−0.52 [−1.60, 0.56]	0%	*p* = 0.34	Not applicable			
FINS-HLWD	−2.26 [−3.00, −1.51]	63%	*p* < 0.00001	Wang et al. (2021)	−1.97 [−2.51, −1.42]	37	*p* < 0.00001
HOMA-IR-DHHL	−0.08 [−0.22,0.06]	0%	*p* = 0.26	Not applicable			
TC-GGQL	−0.57 [−0.99, −0.15]	88%	*p* = 0.008	Fan et al. (2017)	−0.60 [−0.80, −0.40]	0	*p* < 0.00001
				Jin et al. (2019)			
TC-HLEJ	−1.38 [−1.62, −1.14]	70%	*p* < 0.00001	Wang, (2020)	−1.50 [−1.68, −1.33]	0	*p* < 0.00001
TG-GGQL	−0.46 [−0.78, −0.13]	89%	*p* = 0.006	Jin et al. (2019)	−0.26 [−0.38, −0.14]	4	*p* < 0.0001
TG-HLEJ	−1.19 [−1.84, −0.55]	96%	*p* = 0.0003	Wang, (2020)	−1.48 [−1.70, −1.26]	0	*p* < 0.00001
LDL-c-HLEJ	−1.17 [−1.77, −0.57]	94%	*p* = 0.0001	Wang, (2020)	−1.44 [−1.66, −1.23]	0	*p* < 0.00001
LDL-c-HLWD	−0.36 [−0.71, 0.00]	87%	*p* = 0.05	Ji, (2017)	−0.48 [−0.68, −0.28]	36	*p* < 0.00001
HDL-c-DHHL	−0.32 [−0.54, −0.10]	81%	*p* = 0.005	Jin et al. (2019)	−0.45 [−0.59, −0.30]	0	*p* < 0.00001

### 3.6 Publication bias

**FIGURE 5:**
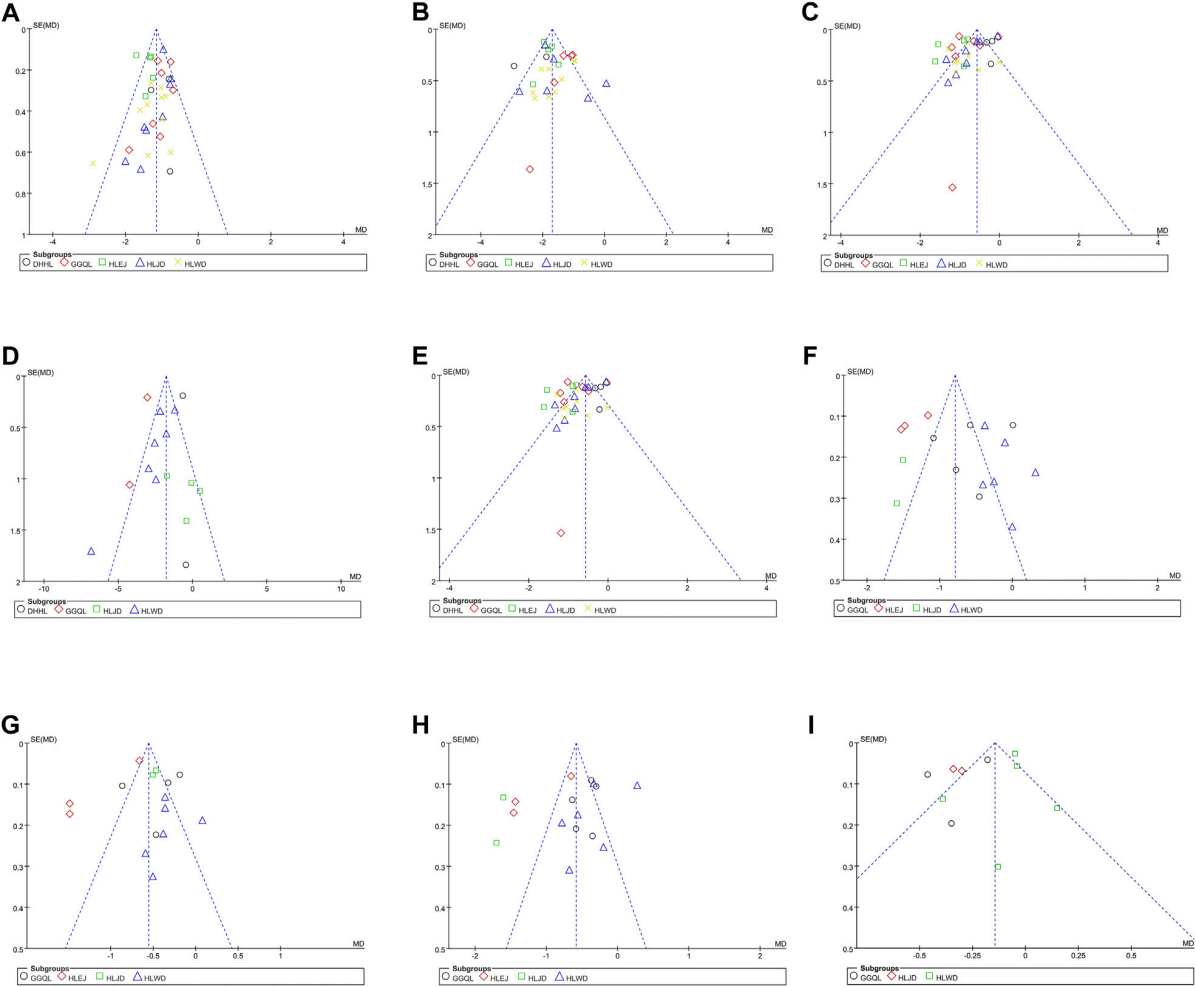
Funnel plots of the trials that compared HLPs plus metformin with metformin. Note: **(A)** Represents FBG; **(B)** Represents 2hPG; **(C)** Represents HbA1c; **(D)** Represents FINS; **(E)** Represents HOMA-IR; **(F)** Represents TC; **(G)** Represents TG; **(H)** Represents LDL-c; **(I)** Represents HDL-c.

**FIGURE 6:**
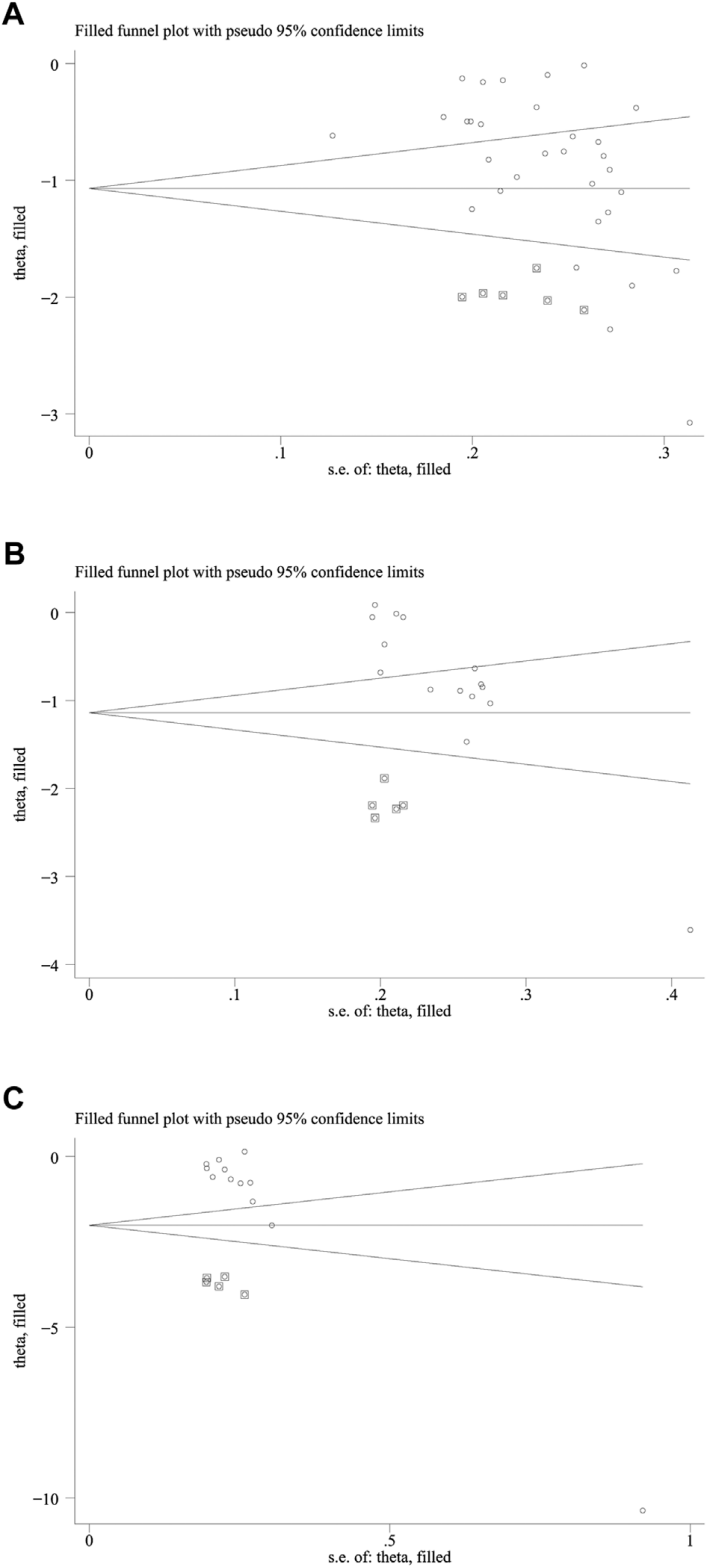
The trim and fill analysis of HbA1c and HOMA-IR. Note: **(A)** Represents the trim and fill analysis of HbA1c; **(B)** Represents the trim and fill analysis of FINS; **(C)** Represents the trim and fill analysis of HOMA-IR. The circle represents the actual estimate and the square represents the theoretical estimate when publication bias does not exist.

As shown in Figure 5, the funnel plots used to evaluate the effectiveness of HLPs in improving FBG are nearly symmetrical, whereas those used to assess the effects of HLPs on 2hPG, HbA1c, FINS, HOMA-IR, TC, TG, LDL-c, and HDL-c are asymmetrical. Therefore, Egger’s test (Stata version 13.0) was also performed to evaluate their publication bias. The Egger’s test used to assess publication bias suggested that *p* > 0.05 in FBG, 2hPG, TC, TG, LDL-c, and HDL-c, whereas *p* < 0.05 in HbA1c, FINS, and HOMA-IR (Table 5). Finally, the trim-and fill-method (Stata version 13.0) was used to evaluate the publication bias of HbA1c and HOMA-IR. In Figure 6A, theoretically missing studies show an adjusted improvement in HbA1c, corresponding to −1.083 MD [95% CI, −1.346 to −0.853], relative to -0.932 MD [95% CI, −1.182 to −0.791]. As shown in Figure 6B, five theoretically missing studies show corrected improvement in FINS, corresponding to 0.275 MD [95% CI, 0.069 to 0.412], compared with -1.144 MD [95% CI, −1.792 to −0.645]. As shown in Figure 6C, five theoretically missing studies show corrected improvement in HOMA-IR, corresponding to 0.141 MD [95% CI, 0.061 to 0.371], compared with −1.142 MD [95% CI, −1.787 to −0.582].

**TABLE 5:** Egger’s publication test of the trials that compared HLPs plus metformin vs. metformin.

Detection indicators	*p* Value
FBG	*p* = 0.325
2hPG	*p* = 0.233
HbA1c	*p* = 0.007
FINS	*p* < 0.001
HOMA-IR	*p* < 0.001
TC	*p* = 0.063
TG	*p* = 0.058
LDL-c	*p* = 0.286
HDL-c	*p* = 0.370

## 4 Discussion

The potential of HLPs to prevent and treat T2DM has been investigated in several studies, and its hypoglycemic mechanism is becoming increasingly apparent. DHHL decoction can regulate the glucose level by activating AMPKα and upregulating the expression of PGC-1α and GLUT4 (Hao et al., 2019). GGQL decoction can enhance glucose metabolism by regulating tryptophan, pantothenic acid, and adenine in IR-HepG2 cells (Chen et al., 2018), as well as improve liver insulin resistance by upregulating SIRT1 expression and reducing FoxO1 acetylation (Sui et al., 2018). HLEJ decoction can exert glucose-lowering and lipid-lowering effects by resisting inflammation and improving insulin resistance (Feng, 2015). HLJD decoction can exert hypolipidemic effects by inhibiting the increased activity of intestinal pancreatic lipase (Zhang et al., 2013) and increasing GLUT4 and PI3K p85 mRNA expression in adipose and skeletal muscle tissues (Chen et al., 2007; Jin et al., 2007). HLWD decoction can effectively treat glycometabolism disorder by repairing the insulin signaling pathway and inhibiting the release of inflammatory cytokines (Li et al., 2016; Chen et al., 2019).

This systematic review and meta-analysis included 33 RCTs involving 2,846 participants. In this study, the included RCTs were rigorously screened and controlled. With regard to quality, the risks of detection bias (33 trials had low risks), attrition bias (32 trials had low risks), reporting bias (24 trials had low risks), and other bias (23 trials had low risks) were generally low, but the risks of selection bias (18 trials had low risks) and performance bias (3 trials had low risks) were generally unclear. Therefore, the methodological quality was considerably moderate. Findings from this study indicate that compared with metformin alone, HLPs combined with metformin is more beneficial for FBG, 2hPG, HAb1c, FINS, HOMA-IR, TC, TG, LDL-c, and, HDL-c, but the improvement of HLPs on GD was not statistically significant.

In this study, treatment with different HLPs exhibited different hypoglycemic and lipid-lowering effects, suggesting that metformin combined with different HLPs may cause variations in medicinal metabolism. This study found that DHHL decoction can improve FBG, 2hPG, HbA1c, and FINS, but does not affect HOMA-IR. In addition, no well-established data are available to analyze the effect of DHHL decoction on TC, TG, LDL-c, and HDL-c. GGQL decoction can improve all blood glucose and blood lipid indicators. HLEJ decoction can improve FBG, 2hPG, HbA1c, TC, TG, and LDL-c, but its role in FINS, HOMA-IR, and HDL-c has not been reported. HLJD decoction can improve FBG, 2hPG, HbA1c, HOMA-IR, TC, TG, LDL-c, and HDL-c, but exerts no effect on FINS. HLWD decoction can improve FBG, 2hPG, HbA1c, FINS, HOMA-IR, and TG, but the improvement in TC, LDL-c, and HDL-c was not statistically significant. Therefore, among all HLPs, GGQL decoction is potentially the most effective prescription for improving T2DM.

The advantages of this study are as follows: 1) In the sensitivity analysis, the difference in prescriptions may be the important source of heterogeneity, so we performed a subgroup analysis in different HLPs. Meanwhile, the overall results exhibited heterogeneity in this study, so we excluded the individual trials that caused heterogeneity, and the heterogeneity was significantly reduced. 2) With regard to publication bias, we used funnel plot, Egger’s test, and trim-and-fill method to evaluate the publication bias. The results of funnel plot and Egger’s test suggest that no publication bias was found in the enhancing effect of HLPs on FBG, 2hPG, TC, TG, LDL-c, and HDL-c. Then the trim-and-fill method was used to further evaluate the publication bias of HbA1c, FINS, and HOMA-IR, which still has important reference significance for the improvement of HbA1c, FINS, and HOMA-IR with HLPs. 3) This study also applied TSA analysis to assess the sample size required and thereby draw reliable conclusions. The sample size of all but one (HLPs for GD) were found sufficient to support this study and thereby draw reliable conclusions. Therefore, the results of this study present high reliability.

The present study also has several limitations: 1) All RCTs included in this study were Chinese, which likely led to geographical bias. Thus, an international collaboration should be conducted to ensure the generalizability of the findings. 2) The methodological quality of the RCTs was low, only half of the RCTs described the allocation concealment and blinding method, which might have led to a nonnegligible risk of bias. Thus, more scientific RCTs with specific randomize allocation details are needed. 3) Different kinds of HLPs vary in their hypoglycemic mechanism of action. Thus, high heterogeneity was observed among different HLPs, limiting the confirmation of the efficacy of HLPs in the treatment of T2DM. 4) Variations in dose in the same prescription are a concern in TCM. Variations in dose may also lead to differences in efficacy, leading to heterogeneity in research. 5) Current evidence shows that GGQL decoction can be potentially used as the optimal complementary approach to regulate glucose and lipid levels, but this finding has yet to be proved. Therefore, more rigorously designed and large-scale RCTs are required to confirm our findings.

## 5 Conclusion

Current evidence from this meta-analysis and systematic review suggests that compared with metformin alone, HLPs provide more benefits for the treatment of T2DM, particularly in FBG, 2hPG, HAb1c, FINS, HOMA-IR, TC, TG, LDL-c, and HDL-c. Due to insufficient data from the included RCTs, the therapeutic effect of HLPs on GD has not been demonstrated, and the findings should be elucidated with caution because of the limitations. Therefore, larger-scale and well-designed RCTs are essential to verify HLPs as a promising candidate treatment for patients with T2DM.

## Data Availability

The original contributions presented in the study are included in the article/Supplementary Material, further inquiries can be directed to the corresponding author.

## Abbreviations

2hPG, 2-h postprandial blood glucose; AIS, accrued information size; CI, confidence intervals; CNKI, China National Knowledge Infrastructure; DHHL, Dahuang huanglian xiexin; FBG, fasting blood glucose; FINS, fasting serum insulin; GD, gastrointestinal dysfunction; GGQL, Gege qinlian; GRADE, Grades of Recommendation Assessment Development and Evaluation; HbA1c, glycosylated hemoglobin type A1c; HDL-c, high density lipoprotein cholesterol; HLEJ, Huanglian ejiao; HLJD, Huanglian jiedu; HLPs, Huanglian prescriptions; HLWD, Huanglian wendan; HOMA-IR, homeostasis model assessment of insulin resistance; LDL-c, low density lipoprotein cholesterol; MD, mean difference; RCTs, randomized controlled trials; RIS, required information size; T2DM, type 2 diabetes mellitus; TC, total cholesterol; TCM, traditional Chinese medicine; TG, triglyceride; TSA, trial sequential analysis.
