# Corrigendum: Systematic review and meta-analysis of *Coptis Chinensis* Franch. -containing traditional Chinese medicine as an adjunct therapy to metformin in the treatment of type 2 diabetes mellitus

**DOI:** 10.3389/fphar.2022.1112418

**Published:** 2023-01-04

**Authors:** Linlin Pan, Xin Zhai, Zhanhui Duan, Kun Xu, Guirong Liu

**Affiliations:** Shandong University of Traditional Chinese Medicine, Jinan, Shandong, China

**Keywords:** *Coptis chinensis* Franch*.*, type 2 diabetes mellitus, systematic review, meta-analysis, Chinese medicine

In the published article, there was an error in the **Funding** statement. Only one funder appeared in the final published article. The correct Funding statement appears below.

Funding

“This work was supported by Qilu Zhiyuan academic school inheritance project (2022-93); Cultivation project of science and technology history of Shandong University of traditional Chinese Medicine (2021KJSKFKT-B03); National Traditional Chinese Medicine Administration Key Discipline Construction Project of Traditional Chinese Medicine National Chinese Medicine Offifice Letter (2012) No. 32”.

We apologize for this error and state that this does not change the scientific conclusions of the article in any way. The original article has been updated.

