# Supplementary figures and images for: Systematic review and meta-analysis of Coptis chinensis Franch.-containing traditional Chinese medicine as an adjunct therapy to metformin in the treatment of type 2 diabetes mellitus
Source: Front Pharmacol. 2022 Sep 8;13:956313. doi: 10.3389/fphar.2022.956313 (PMC9492976; doi:10.3389/fphar.2022.956313)

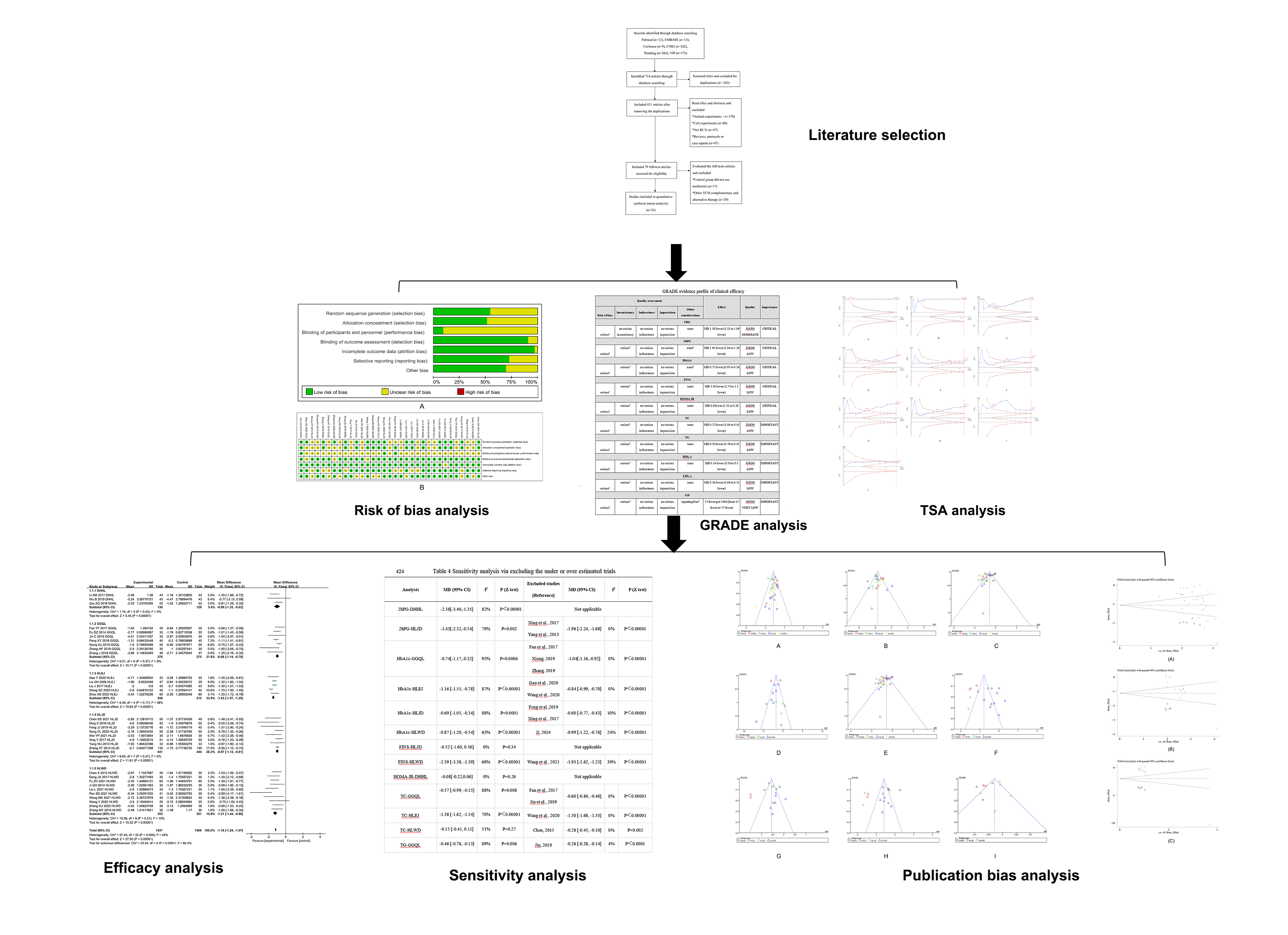

Supplement: Supplementary file 1 [file Image1.TIF]
