# Supplementary material for: Systematic review and meta-analysis of Coptis chinensis Franch.-containing traditional Chinese medicine as an adjunct therapy to metformin in the treatment of type 2 diabetes mellitus
Source: Front Pharmacol. 2022 Sep 8;13:956313. doi: 10.3389/fphar.2022.956313 (PMC9492976; doi:10.3389/fphar.2022.956313)
Supplement: Supplementary file 2 [file Table1.DOC]

| **Pubmed** | | |
| --- | --- | --- |
| Search | Query | Items found |
| #10 (#3 AND #6 AND #9) | Search (((((((((((type 2 diabetes mellitus[Title/Abstract]) OR T2DM[Title/Abstract]) OR impaired fasting glucose[Title/Abstract]) OR impaired glucose tolerance[Title/Abstract]) OR Xiaoke syndrome[Title/Abstract])) OR type 2 diabetes[Title/Abstract])) OR non insulin dependent diabetes mellitus [Title/Abstract])) OR "Diabetes Mellitus, Type 2"[Mesh])) AND ((((((((randomized controlled trial[Title/Abstract]) OR controlled clinical trial[Title/Abstract]) OR random[Title/Abstract]) OR randomly[Title/Abstract]) OR randomized[Title/Abstract]) OR RCT[Title/Abstract])) OR "Random Allocation"[Mesh])) AND (("huanglian" [Supplementary Concept]) OR ((((Glycyrrhiza uralensis Fisch.[Title/Abstract]) OR Huanglian[Title/Abstract]) OR Coptidis Rhizoma[Title/Abstract]) OR Coptis chinensis[Title/Abstract])) | 12 |
| #9 (#7 OR #8) | Search ("huanglian" [Supplementary Concept]) OR (((((Glycyrrhiza uralensis Fisch.[Title/Abstract]) OR Huanglian[Title/Abstract]) OR Coptidis Rhizoma[Title/Abstract]) OR Coptis chinensis[Title/Abstract])OR Rhizoma coptidis[Title/Abstract]) | 1031 |
| #8 | Search (((Glycyrrhiza uralensis Fisch.[Title/Abstract]) OR Huanglian[Title/Abstract]) OR Coptidis Rhizoma[Title/Abstract]) OR Coptis chinensis[Title/Abstract] | 1025 |
| #7 | Search "huanglian" [Supplementary Concept] | 61 |
| #6 (#4 OR #5) | Search (((((((((randomized controlled trial[Title/Abstract]) OR controlled clinical trial[Title/Abstract]) OR random[Title/Abstract]) OR randomly[Title/Abstract]) OR randomized[Title/Abstract]) OR RCT[Title/Abstract])) OR "Random Allocation"[Mesh])) OR placebo[Title/Abstract] | 1169163 |
| #5 | Search (((((randomized controlled trial[Title/Abstract]) OR controlled clinical trial[Title/Abstract]) OR random[Title/Abstract]) OR randomly[Title/Abstract]) OR randomized[Title/Abstract]) OR RCT[Title/Abstract] | 1023821 |
| #4 | Search "Random Allocation"[Mesh] | 102119 |
| #3 (#1 OR #2) | Search ((((((((type 2 diabetes mellitus[Title/Abstract]) OR T2DM[Title/Abstract]) OR impaired fasting glucose[Title/Abstract]) OR impaired glucose tolerance[Title/Abstract]) OR Xiaoke syndrome[Title/Abstract])) OR type 2 diabetes[Title/Abstract])) OR "Diabetes Mellitus, Type 2"[Mesh] | 183766 |
| #2 | Search (((((((type 2 diabetes mellitus[Title/Abstract]) OR T2DM[Title/Abstract]) OR impaired fasting glucose[Title/Abstract]) OR impaired glucose tolerance[Title/Abstract]) OR Xiaoke syndrome[Title/Abstract]) OR type 2 diabetes[Title/Abstract]) OR non insulin dependent diabetes mellitus [Title/Abstract] | 132332 |
| #1 | Search "Diabetes Mellitus, Type 2"[Mesh] | 129045 |

**Embase**

| No. | Query | Results |
| --- | --- | --- |
| #25 | ('non insulin dependent diabetes mellitus'/exp OR 'type 2 diabetes mellitus':ab,ti OR 't2dm':ab,ti OR 'impaired fasting glucose':ab,ti OR 'impaired glucose tolerance':ab,ti OR 'xiaoke':ab,ti OR 'type 2 diabetes':ab,ti) AND ('randomization'/exp OR 'random allocation':ab,ti OR 'randomized controlled trial':ab,ti OR 'controlled clinical trial':ab,ti OR 'random':ab,ti OR 'randomly':ab,ti OR 'randomized':ab,ti OR 'placebo':ab,ti OR 'rct':ab,ti) AND ('huanglian'/exp OR 'glycyrrhiza uralensis fisch.':ab,ti OR 'coptidis rhizoma.':ab,ti OR 'coptis chinensis.':ab,ti OR 'rhizoma coptidis.':ab,ti) | 13 |
| #24 | 'huanglian'/exp OR 'glycyrrhiza uralensis fisch.':ab,ti OR 'coptidis rhizoma.':ab,ti OR 'coptis chinensis.':ab,ti OR 'rhizoma coptidis.':ab,ti | 1447 |
| #23 | 'rhizoma coptidis.':ab,ti | 360 |
| #22 | 'coptis chinensis.':ab,ti | 499 |
| #21 | 'coptidis rhizoma.':ab,ti | 404 |
| #20 | 'glycyrrhiza uralensis fisch.':ab,ti | 209 |
| #19 | 'huanglian'/exp | 42 |
| #18 | 'randomization'/exp OR 'random allocation':ab,ti OR 'randomized controlled trial':ab,ti OR 'controlled clinical trial':ab,ti OR 'random':ab,ti OR 'randomly':ab,ti OR 'randomized':ab,ti OR 'placebo':ab,ti OR 'rct':ab,ti | 1536492 |
| #17 | 'rct':ab,ti | 36074 |
| #16 | 'placebo':ab,ti | 301246 |
| #15 | 'randomized':ab,ti | 726623 |
| #14 | 'randomly':ab,ti | 431988 |
| #13 | 'random':ab,ti | 316220 |
| #12 | 'controlled clinical trial':ab,ti | 18612 |
| #11 | 'randomized controlled trial':ab,ti | 93221 |
| #10 | 'random allocation':ab,ti | 2011 |
| #9 | 'randomization'/exp | 85815 |
| #8 | 'non insulin dependent diabetes mellitus'/exp OR 'type 2 diabetes mellitus':ab,ti OR 't2dm':ab,ti OR 'impaired fasting glucose':ab,ti OR 'impaired glucose tolerance':ab,ti OR 'xiaoke':ab,ti OR 'type 2 diabetes':ab,ti | 298090 |
| #7 | 'type 2 diabetes':ab,ti | 183860 |
| #6 | 'xiaoke':ab,ti | 65 |
| #5 | 'impaired glucose tolerance':ab,ti | 16247 |
| #4 | 'impaired fasting glucose':ab,ti | 5961 |
| #3 | 't2dm':ab,ti | 35156 |
| #2 | 'type 2 diabetes mellitus':ab,ti | 61155 |
| #1 | 'non insulin dependent diabetes mellitus'/exp | 250927 |

**Cochrane Library**

| ID | Search | Results |
| --- | --- | --- |
| #1 | MeSH descriptor: [Diabetes Mellitus, Type 2] explode all trees | 17001 |
| #2 | MeSH descriptor: [Huanglian] explode all trees | 0 |
| #3 | (type 2 diabetes):ti,ab,kw (Word variations have been searched) | 45470 |
| #4 | (type 2 diabetes mellitus):ti,ab,kw (Word variations have been searched) | 38855 |
| #5 | (T2DM):ti,ab,kw (Word variations have been searched) | 5945 |
| #6 | (non insulin dependent diabetes mellitus):ti,ab,kw (Word variations have been searched) | 16726 |
| #7 | (impaired fasting glucose):ti,ab,kw (Word variations have been searched) | 2684 |
| #8 | (impaired glucose tolerance):ti,ab,kw (Word variations have been searched) | 3907 |
| #9 | (Xiaoke):ti,ab,kw (Word variations have been searched) | 41 |
| #10 | (Huanglian):ti,ab,kw (Word variations have been searched) | 35 |
| #11 | (Glycyrrhiza uralensis Fisch.):ti,ab,kw (Word variations have been searched) | 1 |
| #12 | (Coptidis Rhizoma):ti,ab,kw (Word variations have been searched) | 25 |
| #13 | (Coptis chinensis):ti,ab,kw (Word variations have been searched) | 16 |
| #14 | (Rhizoma coptidis):ti,ab,kw (Word variations have been searched) | 25 |
| #15 | #1 OR #3 OR #4 OR #5 OR #6 OR #7 OR #8 OR #9 | 50346 |
| #16 | #2 OR #10 OR #11 OR #12 OR #13 OR #14 | 69 |
| #17 | #15 #16 | 9 |

**CNKI (in the advanced search)**

| ID | Search | Results |
| --- | --- | --- |
| #1 | MeSH: Erxing Tangniaobing OR Xiaoke | 119571 |
| #2 | #1 AND (Huanglian) | 30667 |
| #3 | #2 AND (Suiji duizhao shiyan OR Mangfa OR Shuangmang OR Suiji duizhao OR Anweiji) | 66421 |
| #4 | #3 AND (Dahuang Huanglian OR Gegen Qinlian OR Huanglian Ejiao OR Huanglian Wendan OR Huanglian Jiedu) | 242 |

**Wangfang (in the advanced search)**

| ID | Search | Results |
| --- | --- | --- |
| #1 | MeSH: Erxing Tangniaobing OR Xiaoke | 256384 |
| #2 | #1 AND (Huanglian) | 58739 |
| #3 | #2 AND (Suiji duizhao shiyan OR Mangfa OR Shuangmang OR Suiji duizhao OR Anweiji) | 1774 |
| #4 | #3 AND (Dahuang Huanglian OR Gegen Qinlian OR Huanglian Ejiao OR Huanglian Wendan OR Huanglian Jiedu) | 263 |

**VIP (in the advanced search)**

| ID | Search | Results |
| --- | --- | --- |
| #1 | MeSH: Erxing Tangniaobing OR Xiaoke | 129522 |
| #2 | #1 AND (Huanglian) | 26181 |
| #3 | #2 AND (Suiji duizhao shiyan OR Mangfa OR Shuangmang OR Suiji duizhao OR Anweiji) | 952 |
| #4 | #3 AND (Dahuang Huanglian OR Gegen Qinlian OR Huanglian Ejiao OR Huanglian Wendan OR Huanglian Jiedu) | 175 |
